# High-Sensitivity Defect Inspection for Unpatterned Wafers via Integrating Dark-Field Scattering and Diffraction Phase Microscopy

**DOI:** 10.3390/s26041271

**Published:** 2026-02-15

**Authors:** Xiangchao Zhang, Qianru Zheng, Di Li, Ruifang Ye

**Affiliations:** 1 Jianghuai Advanced Technology Center, Hefei 230088, China; 2 Shanghai Engineering Research Center of Ultra-Precision Optical Manufacturing, College of Future Information Technology, Fudan University, Shanghai 200438, China; 22210720321@m.fudan.edu.cn; 3 Beijing Institute of Aerospace Precision Machinery, Aviation Industry Corporation of China, Beijing 100076, China; lid066@avic.com; 4 College of Mechanical Engineering and Automation, Huaqiao University, Xiamen 361021, China

**Keywords:** wafer inspection, defect detection, dark field scattering, diffraction phase microscopy

## Abstract

To overcome the limitations in the sensitivity and reliability of conventional wafer defect inspection techniques, a novel dual-channel optical inspection system is proposed by combining dark-field scattering with diffraction phase microscopy. Such an integrated system simultaneously acquires dark-field intensity and phase gradient signals arising from wafer defects, enabling comprehensive defect characterization at identical wafer locations while maintaining high sensitivity and high efficiency. Experimental validation using polystyrene particles demonstrates that the system achieves a limit of detection of 60 nm, improves the detecting sensitivity compared to single dark field scattering systems, and maintains the lateral/vertical limit of detection for small-scale defects. These results confirm its potential to meet the high-sensitivity and high-reliability requirements of unpatterned wafer defect inspection for advanced semiconductor manufacturing.

## 1. Introduction

The relentless scaling of semiconductor devices, in alignment with Moore’s Law, has intensified the demand for defect inspection techniques that are capable of detecting nanoscale imperfections on unpatterned wafers [1]. Such defects—often originating during wafer preparation or film deposition—can severely compromise device performance and yield [2]. Optical inspection methods of wafer defects have become the industry standard owing to their non-contact operation, high throughput, and compatibility with large-area substrates [3].

Among these methods, bright-field microscopy based on the variations of light reflected from or transmitted through the samples under inspection has been widely deployed for defect identification. However, as the sizes of critical defects shrink below the illumination wavelength, bright-field techniques suffer from deteriorating signal-to-noise ratios and insufficient contrast [4,5]. Consequently, dark-field scattering (DFS) microscopy has emerged as a powerful alternative. By detecting laterally scattered light from micro-scaled defects, DFS enhances sensitivity to surface features such as nanoparticles and shallow scratches [6]. Commercial systems from industry leaders (e.g., KLA’s Puma and Surfscan platforms) leverage DFS principles to achieve remarkable detection capabilities.

Despite these advances, DFS exhibits inherent limitations. First, its sensitivity is strongly influenced by illumination geometry and defect orientation, often resulting in weak signals for certain defect types [7]. Second, DFS provides limited depth resolution, hampering the characterization of embedded or three-dimensional defects [8,9]. Numerous research efforts have sought to mitigate these issues. For instance, laser-scanning dark-field confocal systems have improved scanning efficiency, but introduce false detections due to photomultiplier gain instability [10]. Time-delay integration (TDI) sensors have been incorporated to enhance signal-to-noise ratio, yet remain susceptible to imaging aberrations and vibration-induced artifacts [11]. Computational approaches by combining DFS with machine learning show promise in nanoparticle identification but lack real-time processing capabilities [12]. Other methods, such as dynamic light scattering [13] or active focus stabilization [14], have niche applicability but face challenges in terms of integration or cost-effectiveness. Hybrid illumination systems, e.g., bright-field with dark-field, have been proposed for mirror-surface inspection, though their scalability for full-wafer applications remains limited [15].

To address these challenges, we propose a dual-channel optical inspection system that integrates DFS with diffraction phase microscopy (DPM). This configuration simultaneously acquires scattered intensity and quantitative phase gradient information, enabling multi-parameter defect characterization at identical spatial locations. The system maintains high sensitivity and operational robustness. In this paper, the optical design, operational principles, and experimental validation of the system are presented, demonstrating its capability for high-sensitivity defect inspection for unpatterned wafers.

## 2. Inspection Principle and Methodology

### 2.1. Principle and System Design

**Figure 1:**
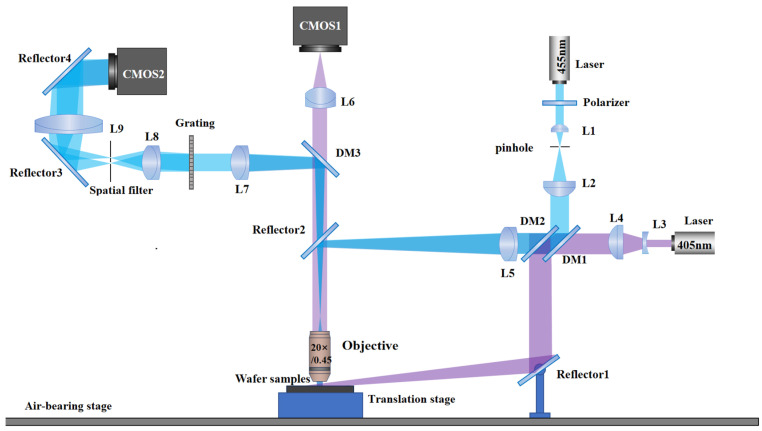
Schematic diagram of dual channel optical inspection system.

The dual-channel optical inspection system is composed of DFS and DPM, as shown in Figure 1. Here, the purple ray path represents the DFS imaging module, and the blue ray path corresponds to the DPM imaging module. In the DFS channel, a 405 nm laser beam is expanded by a Galilean telescope system (lenses L3, L4), transmits through a short-pass dichroic mirror DM1 (transmits 405 nm/reflects 455 nm), and is reflected by a long-pass dichroic mirror DM2 (transmits 455 nm/reflects 405 nm). The reflected beam obliquely illuminates the sample via an adjustable mirror M1 to induce scattering. The scattered light collected by the objective is redirected by a mirror M2 and transmits through a short-pass dichroic mirror DM3 (transmits 405 nm/reflects 455 nm), ultimately imaged onto the camera CMOS1 by the tube lens L6. In the DPM channel, a 455 nm laser passes through a polarizer and a beam-expanding collimation system (achromatic lenses L1, L2 with a pinhole), is reflected by DM1 and transmits through DM2. The beam is focused by a lens L5 onto the objective’s back focal plane to form collimated vertical illumination. The light collected by the objective is redirected by M2 and reflected by DM3 to a tube lens L7. At the focal plane of L7, a diffraction grating generates multiple diffraction orders. The diffracted beams pass through a lens L8, whose focal plane is aligned with the grating, to a spatial filter, where only zero-order and first-order beams are retained. These dual beams propagate through a lens L9, which forms a 4f system with L8, for spatial filtering and magnification, and finally form an interference pattern on the camera CMOS2 via reflector M4. A 4f system is a Fourier optics architecture with two identical focal-length lenses in confocal alignment. L8 performs a Fourier transform of the sample’s optical field to a spectrum/pupil plane, and L9 an inverse transform to reconstruct the image. It enables spatial filtering, pupil engineering, aberration correction, and telecentric scanning, which is critical for high-resolution microscopic imaging. A sample translation stage enables the transversal scanning on the sample for both channels.

In the integration process of the dual-channel optical system, to realize the effective separation of the 405 nm laser and 455 nm laser in the two imaging modes and avoid mutual interference between them, multiple dichroic mirrors are introduced in the system design. A dichroic mirror can realize selective reflection or transmission according to the different light wavelengths, realizing the optical separation of the two laser beams under the common optical path configuration, so that the two imaging modes can operate independently without interference with each other. In order to further improve the compactness and integration of the system, multiple planar mirrors are used to fold the optical path. At the same time, the two channels share the same objective lens to achieve simultaneous imaging and comparative analysis of the same position at the same sample. The position of the objective lens is precisely controlled by a mechanical adjustment frame to ensure the strict coincidence of the focal plane of the dual-channel optical system, and alignment of the optical axes of the two channels is implemented by using a standard calibration artefact. Finally, the dual-mode synchronous imaging in the same observation area is realized.

### 2.2. Dark-Field Scattering Technique

DFS technology leverages the interaction between incident light and nanoscale surface defects or irregularities [16]. When light illuminates a wafer, sub-wavelength defects induce scattering, which behaves differently from the background light transmitted through or reflected from a normal sample area. By selectively capturing scattered light at specific angles while suppressing background light, dark-field microscopy can significantly enhance image contrast and improve the limit of detection compared to bright field imaging.

The amount of scattered light caused by particle-like defects can be measured by a relative size parameter *σ*, which can be expressed as

(1) $$\sigma =\frac{2\pi nr}{\lambda }$$

where *r* is the radius of defect, *λ* is the light wavelength, and *n* is the refractive index of the surrounding medium. When *σ* ≤ 1, i.e., when the defect size is much smaller than the incident wavelength, the scattering behavior can be described using the quasi-static approximation. According to the Rayleigh scattering theory, the scattered light intensity is inversely proportional to the fourth power of the incident wavelength [17]. Under otherwise identical conditions, the scattered intensity depends solely on the scattering angle $\theta_{s}$,

(2) $$I(\theta_{s})=\frac{\pi^{4}d^{6}}{4\lambda^{4}l^{2}}(\frac{{n^{2}}_{\mathrm{sph}}-1}{{n^{2}}_{\mathrm{sph}}+2}{)}^{2}(1+\cos^{2}\theta_{s})I_{0}$$

In the formula, *I* is the intensity of scattered light, *I*_0_ is the intensity of incident light, *n*_sph_ is the refractive index of defect particles, *l* is the distance from the detector to the scattering object, and *d* is the diameter of the particle being tested.

In the DFS system, Equation (2) indicates that a short-wavelength laser is preferred to enhance scattering. Accordingly, this study employs a laser with a 405 nm wavelength, which increases the Rayleigh scattering intensity by approximately 2.8 times with respect to a visible wavelength of 532 nm and offers remarkable stability and interference resistance.

When selecting the objective lens, a higher numerical aperture increases the angular range of light capture, which in turn improves the detecting sensitivity to weak signals and enhances imaging contrast, while an adequate working distance accommodates oblique illumination and the integration of additional optical components. Moreover, the objective must be compatible with the DPM optical path to facilitate multimodal imaging. Based on these considerations, a flat-field, semi-achromatic dark-field objective with 20× magnification and a 0.45 NA is employed, paired with a tube lens with a focal lens of 200 mm. The field of view is determined by *L*_0_ = *L*/*M*. Here *L* denotes the size of the camera sensor’s photosensitive area. The system achieves a field of view of 622.1 μm × 491.5 μm.

**Figure 2:**
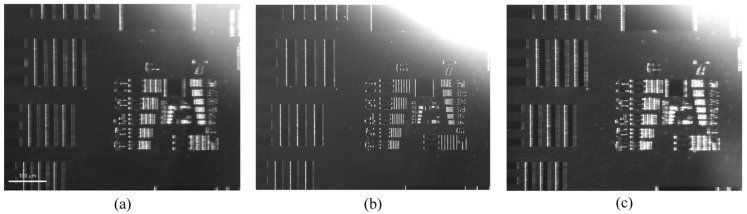
DFS imaging under different incidence angles: (**a**) 30°; (**b**) 45°; (**c**) 60°.

Figure 2 illustrates the imaging results obtained under varying incident angles while maintaining a constant focal distance. As indicated in Figure 2b, the system exhibits a remarkable capability for producing clear images. Figure 2a,b indicates that the intensity of defect signals detected in the DFS varies remarkably with the incidence direction. Even under the optimal focusing conditions, image quality is still influenced by the contrast between background illumination and scattered light. These findings underscore the necessity of a dual-channel optical design to enhance imaging contrast and ensure reliable defect detection.

### 2.3. Diffraction Phase Microscopy Technique

**Figure 3:**
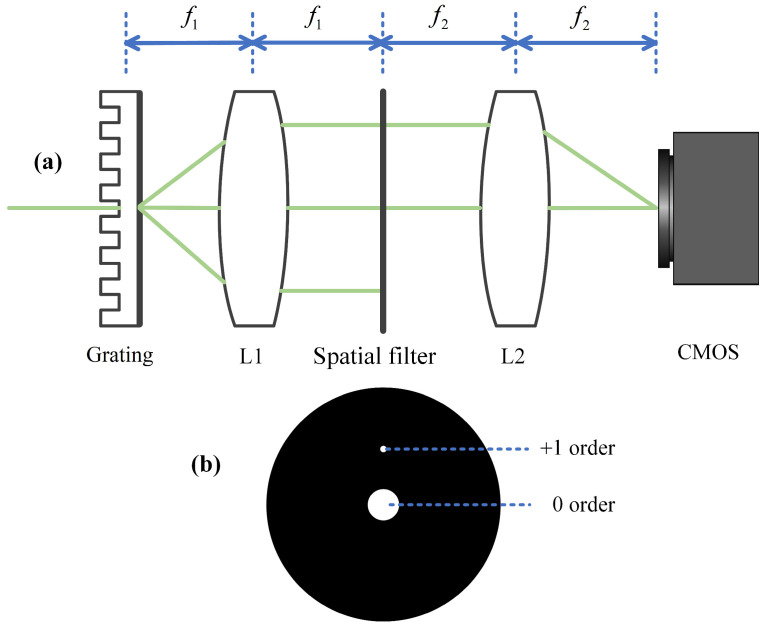
Schematic diagram of diffraction phase microscopy: (**a**) 4f system; (**b**) Spatial filter.

The core configuration of the DPM system is illustrated in Figure 3 [18]. After the sample is imaged through a magnification imaging system, a transmission grating is allocated on the image plane. The beam is separated into several diffraction terms, each propagating along different directions. A 4f optical system is utilized, in which a spatial filter is positioned at the back focal plane of lens L1. This filter allows the 0-order diffraction beam to pass through, while the +1 order beam is filtered via a pinhole, retaining only the DC component and effectively blocking higher-order spatial frequencies. The filtered first-order beam subsequently interferes with the 0-order beam at the back focal plane of lens L2. The resulting interference pattern is recorded by a CMOS camera, thereby enabling effective reconstruction of the sample’s quantitative phase information based on the theory of off-axis digital holography [19].

In the DPM system, the selection of an appropriate grating groove density and spatial filter geometry is critical for achieving spectral decoupling and obtaining high-quality interference images. According to the Nyquist sampling theorem, each diffraction spot must span at least two grating periods to avoid aliasing. The grating constant determines the angular separation between the reference and object beams, thereby ensuring successful phase retrieval in the carrier-frequency interferogram. In addition, the Abbe diffraction limit establishes the relationship between the lateral resolution and the numerical aperture *NA*_obj_ of the objective lens $\rho =\frac{\lambda }{2NA_{\mathrm{obj}}}$. The interference spectrum consists of three lobes. The central lobe with a radius of 2*kNA*_obj_ carries the primary signal, while side lobes with a bandwidth of *kNA*_obj_ may result from environmental disturbances. To extract the phase information associated with the sample, the +1 order component must be fully separated from the central lobe, requiring the carrier frequency *β* to meet specific condition *β* = 2π*M*_obj_/Λ ≥ 3*kNA*_obj_. Here *M*_obj_ is the objective magnification and Λ is the grating constant. Then, the requirement on the grating constant turns out to be $\Lambda \leq \frac{\lambda M_{\mathrm{obj}}}{3NA_{\mathrm{obj}}}$.

A transmission grating with a groove density of 300 lp/mm is used to ensure complete separation of the +1 order component from the central lobe. However, the high line density increases the angular separation between reference and object beams. To prevent over-dense fringes that could hinder phase demodulation, the 4f system’s magnification must be appropriately set. Additionally, to avoid spectral aliasing, the CMOS camera’s spatial sampling rate *k_s_* must meet the following conditions

(3) $$k_{s}\geq sk_{\max}=2(\beta +kNA_{\mathrm{obj}})$$

To get the magnification of the 4f system, *M*_4*f*_ can be rewritten as

(4) $$k_{s}=\frac{2\pi }{a}M_{\mathrm{obj}}\left|M_{4f}\right|\geq 2(\beta +kNA_{\mathrm{obj}})$$

Thus, we can obtain

(5) $$\left|M_{4f}\right|\geq 2a(\frac{1}{\Lambda }+\frac{NA_{\mathrm{obj}}}{\lambda \cdot M_{\mathrm{obj}}})$$

The diffraction angle of the reference light is determined by the grating equation sin*θ* = *mλ*/Λ (with *m* = 1 for +1 order diffraction), and the distance from the +1 order aperture to the filter center is Δ*x* = *f*_1_tan*θ*. To balance the intensities of the two interfering beams so as to enhance the contrast of the interference patter, the +1 order light is filtered through a pinhole, while the zero-order component passes through a circular window. The diameter of the pinhole is related to the Abbe spot size *ρ* = 1.22*λf*_1_/*D*, with *λ* denoting the wavelength and *D* the aperture of the lens L1. While reducing the pinhole size improves uniformity, an excessively small pinhole weakens reference intensity and lowers fringe contrast, necessitating a balance. The level-0 circular window must be large enough to transmit the object’s maximum spatial frequency, which is determined by the objective’s resolution. The spatial position corresponding to this maximum frequency at the spatial filter is given by

(6) $$D_{0}\geq \frac{f_{1}NA_{\mathrm{obj}}}{0.61M_{\mathrm{obj}}}$$

Additionally, the spatial filter should be as thin as possible and blackened to minimize secondary reflections.

### 2.4. Diffractive Reconstruction Based on the Fractional Fourier Transform

DFS imaging can directly obtain high-contrast grayscale images, while the phase analysis of DPM faces inherent limitations in terms of extraction accuracy and noise suppression. To this end, a phase retrieval algorithm is proposed based on the fractional Fourier transform (FrFT). Unlike the regular Fourier transform (FT), the FrFT adapts by varying its order *p*, thereby establishing a strict mapping with the propagation distance *z* for defocus compensation [20]:

(7) $$F^{p}[f(x)](u)=\int_{-\infty }^{+\infty }f(x)K_{\alpha }(x,u)dx$$

Here, $F^{p}$ is a FrFT of order *p*, *f*(*x*) is the input signal, and *K_α_*(*x,u*) is the kernel function of FrFT defined as

(8) $$K_{p}(x,u)=A_{\alpha }\exp\left\{i\pi (x^{2}\cot\alpha -2xu\csc\alpha +u^{2}\cot\alpha )\right\}$$

In the formula, *α* = π*p*/2 is the transformation order, *p* is the fractional order, and $A_{\alpha }=\sqrt{1-i\cot\alpha }$ is the normalization constant.

Diffraction propagation and FrFT are linked by the standard Fresnel diffraction integral:

(9) $$U_{z}(x,y)=\frac{e^{ikz}}{i\lambda z}\iint U_{0}(x^{\prime },y^{\prime })\exp\{\frac{ik}{2z}[{\left(x-x^{\prime }\right)}^{2}+{\left(y-y^{\prime }\right)}^{2}]\}dx^{\prime }dy^{\prime }$$

In the equation, *U_z_*(*x*, *y*) is the complex amplitude at the observation point (*x*, *y*) after propagating a distance *z*, and *U*_0_(*x*′, *y*′) is the complex amplitude of the incident wave at the source point (*x*′, *y*′).

When the propagation distance *z* and the order *p* of FrFT satisfy a relation cot*α* = *λz*/*f*^2^, the Fresnel diffraction can be expressed as

(10) $$\left\{\begin{array}{l} U_{z}(x)=e^{ikz}F^{p}[U_{0}(x^{\prime })]\\ p=2\arcsin(\lambda z/f)/\pi \end{array}\right.$$

where *f* is the focal length. As a result, FrFT enables a continuous transition of *p* to precisely describe diffraction at an arbitrary propagation distance *z*. Notably, when *p* = 1, FrFT reduces to FT, confirming its theoretical inclusiveness. FT corresponds to the Fraunhofer diffraction model, which assumes an infinite propagation distance *z*. However, in practical optical systems, *z* is always finite, making FrFT a more precise and rigorous approach for modeling diffraction under realistic conditions. By optimizing the transformation order *p* and fractional parameters, the directional enhancement of sub-wavelength phase gradient features induced by defects can be achieved.

**Figure 4:**
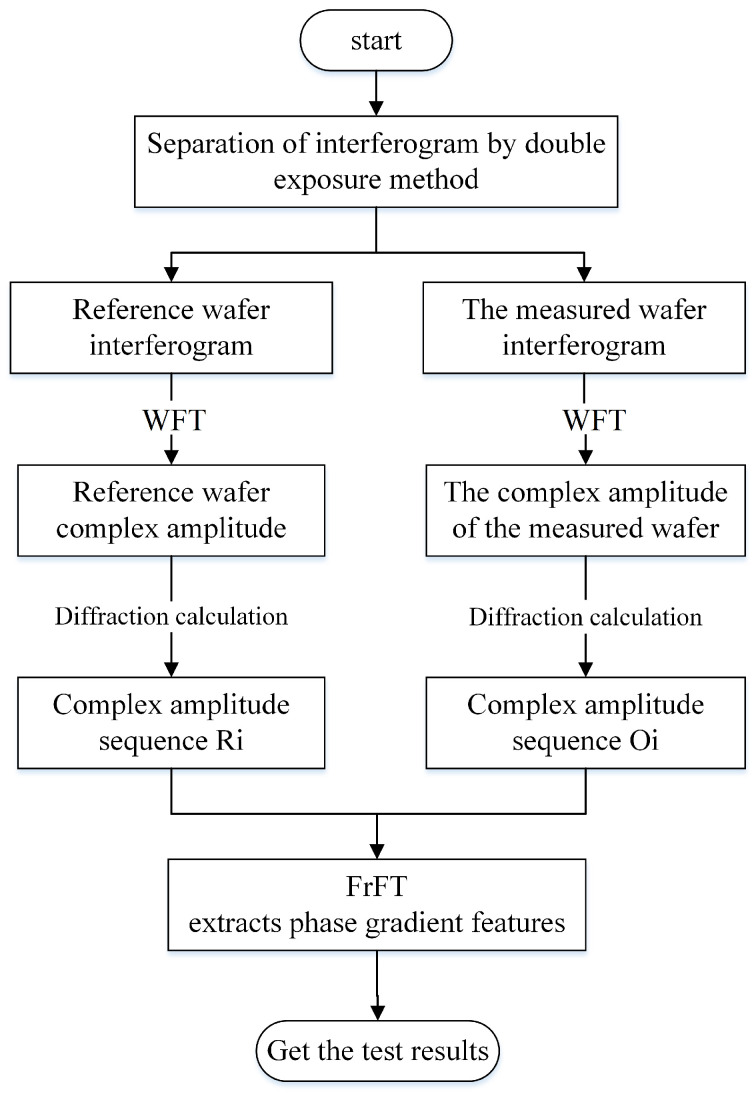
Flowchart of the defect detection method based on FrFT.

The procedure of the defect detection method based on the FrFT is shown in Figure 4.

The interferogram of a reference is captured and stored first. Then, the interference pattern associated with each testing wafer is subsequently captured and superposed with the reference interferogram. Based on the theory of the dual-exposure interferometric measurement [21], the complex amplitude of the testing wafer can be reconstructed using the windowed Fourier transform (WFT) algorithm by designing a weighting function *W*(*u*,*v*), which can effectively suppress speckle noise. The improvement in the signal-to-noise ratio can be expressed as:

$$SNR_{out}=\frac{\sigma_{s}^{2}}{\sigma_{n}^{2}}\cdot \frac{1}{1-\exp(-\tau_{c}/\tau_{0})}$$

where *σ_s_* and *σ_n_* are the variances of the signal and noise, respectively, *τ_c_* is the coherence time, and *τ_r_* is the system response time.

The weighting function in the WFT should be designed according to the characteristics of the actual image. Due to its smoothness, the Gaussian function can effectively reduce the influence of high-frequency noise in the frequency domain while preserving the low-frequency information and structural features of the image. Therefore, it achieves noise suppression in complex amplitude recovery:

$$SNR_{\mathrm{out}}=\frac{\sigma_{s}^{2}}{\sigma_{n}^{2}}\cdot \frac{1}{1-\exp(-\tau_{c}/\tau_{0})}$$

where *σ* is the standard deviation that controls the smoothness of the Gaussian function. By adjusting *σ*, the frequency-domain response of the weighted filter can be controlled, so that low-frequency information is enhanced and high-frequency noise is suppressed.

In order to further enhance the limit of detection, two complex amplitudes are reconstructed in-focus and out-of-focus, respectively. Micro-scaled defects can be straightforwardly identified from the difference of these two amplitudes, mainly according to the phase difference.

## 3. Experimental Results and Discussion

### 3.1. System Setup

**Figure 5:**
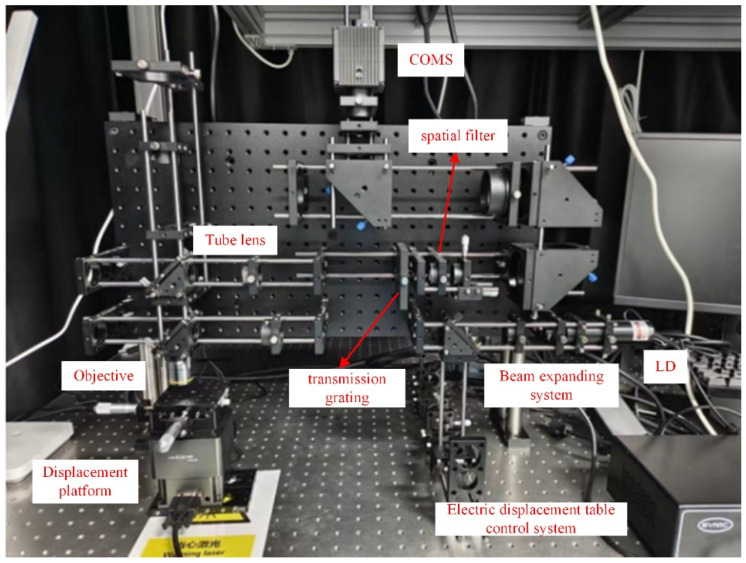
Actual dual-channel inspection system.

An integrated dual-channel inspection system is built, as shown in Figure 5, and the specifications of some key elements are listed in Table 1. Due to the compact arrangement of some optical components, only the main key components are marked in the figure. The overall magnification of the DPM system is 200×, with a field of view of 66.6 μm × 66.6 μm. According to Equation (6), $D_{0}\geq \frac{100\times 0.45}{0.61\times 20}=3.69$ mm. The pinhole distance of the spatial filter is determined as 4.13 mm.

**Figure 6:**
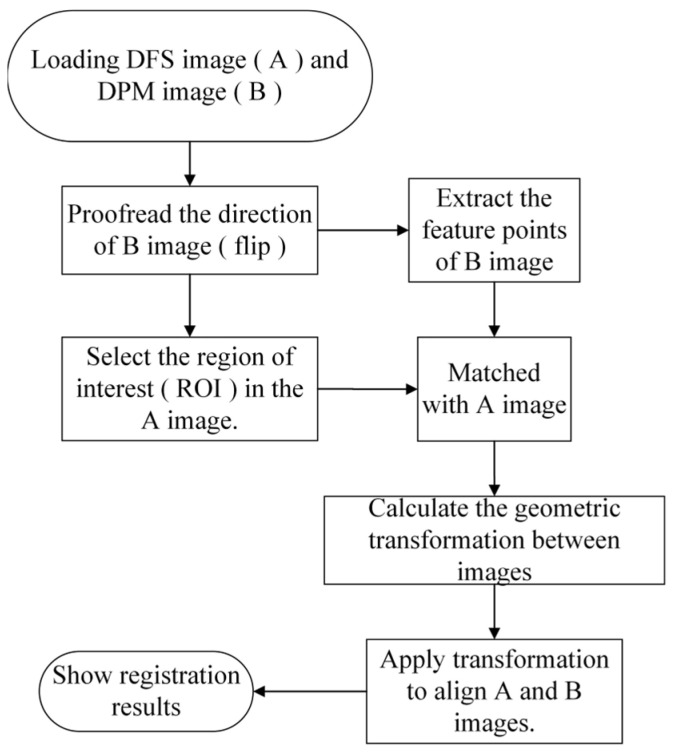
Image alignment process of dual-channel inspection.

The dual-channel optical imaging system collects DFS images and DPM images through independent optical paths and image sensors, respectively. The resulting two images have differences in the lateral axes and amplifications; thus, they need to be aligned. The overall alignment process is shown in Figure 6.

**Figure 7:**
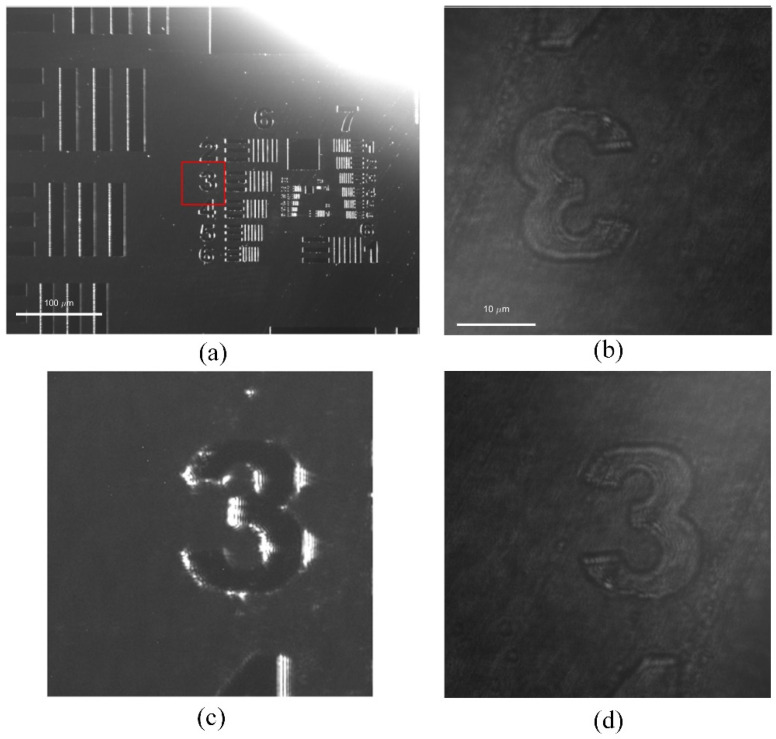
Alignment of images of a resolution plate: (**a**) DFS image; (**b**) DPM image; (**c**,**d**) Part of alignment results of DFS and DPM images.

Before implementing image alignment, a series of preprocessing steps must be carried out to ensure alignment accuracy. First, due to the mirror symmetry between the DFS and DPM images along both the horizontal and vertical directions, the DPM images must undergo horizontal and vertical flipping. Moreover, considering the significant difference in the field of view (FOV) between the DFS and DPM systems, an initial Region of Interest (ROI) should be selected manually or automatically from the DFS image, corresponding to the FOV of the DPM image. Image scaling and feature matching are then performed within this ROI to improve both computational accuracy and efficiency. Effective corner points are identified using the Harris operator $M=\sum_{x,y}\omega (x,y)\left[\begin{array}{l} \begin{array}{cc} I_{x}^{2} & I_{x}I_{y} \end{array}\\ \begin{array}{cc} I_{x}I_{y} & I_{y}^{2} \end{array} \end{array}\right]$, where *ω*(*x*, *y*) denotes the weighting coefficients indicating the ROI, and *I_x_* and *I_y_* denote the partial differences along the x and y directions of the image intensity. A rough alignment is realized by matching some recognized salient feature points, and then a fine alignment is implemented using the Speeded-Up Robust Features (SURF) algorithm, which is an improved version of the Scale-Invariant Feature Transform method. In this method, the affine transformation parameters between the two images are robustly estimated. If the number of matched feature points is insufficient, several control points can be selected manually in turn to construct an affine model, ensuring that registration can also be achieved in weakly textured regions. Finally, the DPM image is transformed into the coordinate system of the DFS image to realize image alignment. This SURF method possesses robustness about the affine transform and illumination variations as well, and the registration accuracy can achieve half a pixel. The random sample consensus algorithm is employed to perform robust filtering on the initial matching set, so as to identify the inlier set with the highest geometric consistency. Ultimately, spatial alignment between the DFS and DPM images is achieved, as illustrated in Figure 7. It is worth mentioning that the transform between two systems is specified using the images associated with a USAF 1951 resolution plate, which contains salient features. While in practical measurement of a wafer, the transformation is directly conducted by using the transformation coefficients determined before.

### 3.2. Testing of Polystyrene Latex of Different Sizes

**Figure 8:**
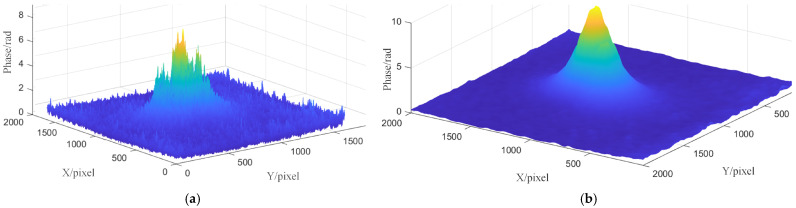
Testing results of 10 μm PSL particle of DPM: (**a**) FT; (**b**) FrFT.

In order to assess the detecting ability of the system, wafer samples containing polystyrene latex (PSL) of different sizes are adopted. The concentration of PSL suspension is 2.5%. Taking PSL of 1 μm as an example, 1 mL suspension contains about 40 billion particles. In order to avoid the aggregation of particles, the suspension is diluted 500 times in ultrapure water, and then uniformly dispersed to a 2-inch polished silicon wafer using a spray bottle. The roughness of the silicon wafer is less than 0.5 nm, the flatness TIR is less than 3 μm, and the warpage TTV is less than 10 μm. All sample preparation and testing are carried out in the cleanroom. The test results of 10 μm size particles are shown in Figure 8.

According to the experimental results, it can be seen that the phase restoring results of FT suffer from strong noise interference, resulting in obvious background noise and artifacts. In addition, two additional false peaks appear around the dominant peak where the defect is located, which implies that the FT method has limitations in terms of noise suppression and signal separation, which may lead to false inspection.

On the contrary, in the phase retrieval results of FrFT, the phase peak corresponding to the defect is clear and single, and no additional false peak appears, which demonstrates the advantages of this method on the robustness against noise and remarkable limit of detection.

**Figure 9:**
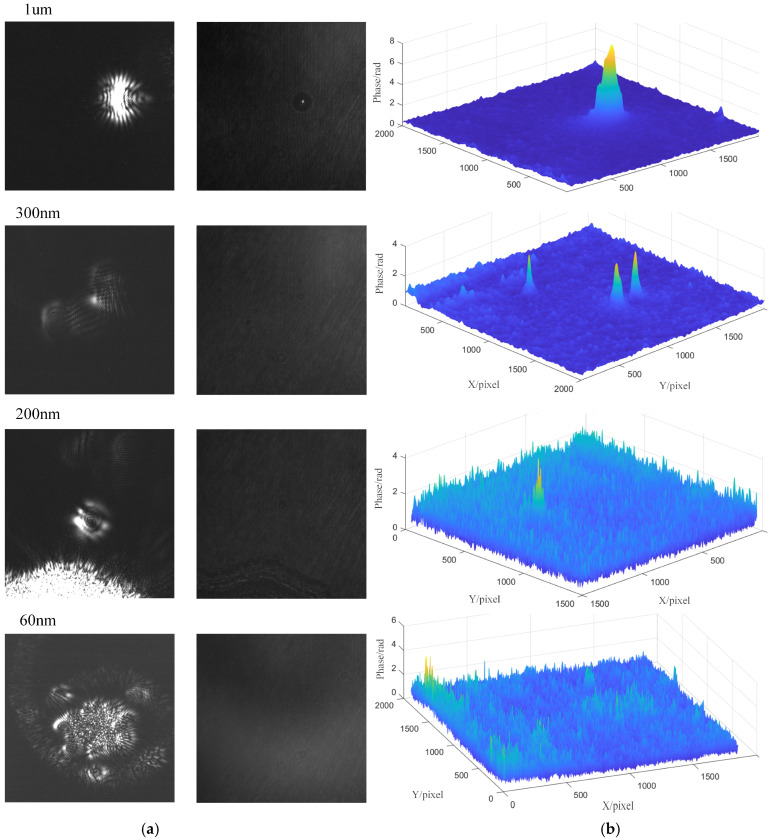
Test results of PSL dual-channel systems with different particle sizes: (**a**) alignment map of the DFS system; (**b**) DPM phase restoring results.

Further experiments are conducted on detecting smaller defects. The detection scale is progressively reduced to a subwavelength level. As shown in Figure 9, four sets of experimental data with defect sizes ranging from 1 μm to 60 nm are selected. Considering the relatively low brightness of the original DPM images, only the phase reconstruction results from the DPM images using the FrFT method are presented here. This approach avoids redundant descriptions and image repetition, while highlighting the capability of detecting defects across multiple scales. When the particle is smaller than the lateral resolution of the system, the phase detected by the DPM system cannot correctly identify the PSL particles. It can be seen that in the DFS image with a large field of view, the initial positioning of the defect can be carried out, and then combined with the DPM image. Then, the fine morphology of the defect can be further obtained.

On the one hand, the comparative analysis of dual-channel imaging can effectively distinguish defects from external pollutants such as specular dust and water stains, thereby reducing the misjudgment rate and improving the limit of detection. On the other hand, DPM can be used for the preliminary determination of defect size and provide morphological information of defects, which lays a foundation for further defect classification and analysis. The DPM system exhibits superior performance in defect detection, providing clearer visualization of defect size, morphology, and structural characteristics. In contrast, within the same size range, DFS system shows reduced imaging clarity and limited recognition capability, offering only the presence of information about defects without quantitatively describing their morphological features. Notably, in the testing of 300 nm particles, although the DFS system is able to detect the presence of defects, it still suffers from image artifacts such as ghost images. The integration of the DPM system effectively mitigated these limitations, enabling more reliable defect characterization. Therefore, compared with the standalone DFS system, the dual-channel detection approach significantly enhances both the sensitivity and reliability of defect inspection.

However, with the further decrease of the defect size, the imaging contrast and phase restoring accuracy of DPM are gradually disturbed by noise, especially when the defect size is reduced to 60 nm. In this case, the DFS system can still maintain good imaging contrast and effectively detect small particles by virtue of its high limit of detection for small defects. Therefore, when the phase retrieval results are disturbed by noise and the defect characteristics are difficult to obtain, the DFS system can still provide good results.

## 4. Conclusions

In this paper, a dual-channel optical imaging inspection system combining dark field scattering and diffraction phase microscopy is proposed to meet the high sensitivity and high reliability requirements in wafer defect inspection. The system realizes multi-modal image acquisition through optimized system design and image alignment. After restoring the phase map of the carrier frequency component based on the fractional Fourier transform, a cooperative inspection of both technologies is successfully realized. The experimental results show that the system can maintain high lateral and vertical sensitivity. In addition, the limit of detection of the system achieves 60 nm, which verifies the feasibility and advantages of the proposed method for practical applications in the semiconductor field.

## Figures and Tables

**Table 1:** Component parameters of the diffraction phase microscope.

Key Element Specification	Value
grating constant	300 lp/mm
4f system magnification	10×
magnification of objective	20×
numerical aperture of objective	0.45
camera pixel size	6.5 μm
exposure time	2 ms
pinhole distance of spatial filter	4.13 mm
grade 1 pinhole diameter	5 μm
focal length of L1	100 mm
0 circular window diameter	2 mm

## Data Availability

Data underlying the results presented in this paper are not publicly available at this time but may be obtained from the authors upon reasonable request.
